# Exclusive breast feeding is the strongest predictor of infant survival in Northwest Ethiopia: a longitudinal study

**DOI:** 10.1186/s41043-015-0007-z

**Published:** 2015-05-01

**Authors:** Gashaw Andargie Biks, Yemane Berhane, Alemayehu Worku, Yigzaw Kebede Gete

**Affiliations:** 1 Institute of public Health, College of Medicine and Health sciences, University of Gondar, P.O.Box 196, Gondar, Ethiopia; 2 Addis Continental Institute of Public Health, Addis Ababa, Ethiopia; 3 School of Public Health, Addis Ababa University, Addis Ababa, Ethiopia

**Keywords:** Infant mortality, Poisson regression, Kaplan-Meier Survival estimates, Longitudinal study design

## Abstract

Despite the overall national success in reducing infant mortality rate in Ethiopia, infant mortality rate is still high in northwest Amhara region. This study is conducted in one of the high mortality areas with the aim of identifying risk factors that are associated with infant mortality in Northwest Amhara Region, Ethiopia. A prospective open cohort study involving 1752 infants (1472.4518 person years of follow-up) was undertaken from November 2009 to August 2011. Kaplan-Meier Survival analysis was used to estimate infant mortality rate. Risk factors associated with infant mortality were assessed using multivariate Poisson regression. The overall infant mortality rate was 88 per 1000 person-years (95% CI: 74.3, 104.9). After controlling other important predictors in multivariate Poisson regression, infants not exclusively breastfed [IRR = 7.86, 95% CI: (5.11, 12.10), )], breast milk initiated after 24 hours of birth [IRR = 4.84,95% CI: (2.94,7.99)], mothers not washing hands with soap after visiting toilet and before feeding child [IRR = 4.61, 95% CI: (2.24, 9.48)], being rural residents [IRR = 2.33, 95% CI: (1.12, 4.88)], infants born within 24 months for the previous birth [IRR = 2.79, 95%CI: (1.88, 4.15)], have increased the risk of infant mortality. In conclusion, exclusive breast feeding is the strongest predictor of infant survival in this predominantly rural setting where hygienic standards are poor. Supporting mothers to exclusively breast feeding which is cost effective, safe and feasible strategy, can help reduce infant mortality in the study setting.

## Background

Infant mortality rate (IMR) is one of the most sensitive and commonly used indicators of social and economic developments of a population [1-3]. Despite the significant progress made in improving child survival in recent years at national levels, infant mortality remains high in some localities in Sub- Saharan Africa [4]. Although Ethiopia has made remarkable progress in reducing the national IMR state level, variations are obvious in addition to the significantly higher infant mortality rate observed in rural compared to urban [4,5]. The Amhara regional state is one of the regions in the country where infant mortality rate is (the) highest, 76/1000 live births [5].

Previous studies have reported several predictors of infant survival, including place of residence [6], maternal illiteracy [7,8], maternal marital status [9,10], and birth interval for the index child, time of initiation of breast milk, exclusive breastfeeding and use of family planning methods [11-14].

Early initiation of breast milk within an hour and exclusive breastfeeding in the first six months of a child’s life is critical for infant survival. Exclusive breastfeeding reduces the risk of diarrheal, pneumonia and other infectious diseases [13,15-17]. Since infant survival is affected by many factors, understanding which factors are important in a particular context is very important in achieving the Millennium Development Goal (MDG) 4, which aims to reduce under five mortality by two-thirds by 2015 in all regions as well as in the country. The objective of this study is, therefore, to identify major predictors for infant survival in the study area.

## Methods

The study was conducted at Dabat Demographic and Health Surveillance System (DHSS) located in the northwestern part of Ethiopia. The study area is predominantly rural and residents largely depend on subsistence farming. A district hospital, two health centers, three health stations, and twenty-nine health posts provide basic health care for the community [18,19]. The district altitude ranges from 1000 to 2500 meters above sea level.

The Dabat DHSS covers ten randomly selected kebeles/neighborhoods (three urban and seven rural kebeles), located in three ecological zones (high land, mid land, and low land). About 3.0% of the populations are infants. Each individual and households in the DHSS has a unique identifier and are visited by a trained interviewer every six months to record vital events as well as pregnancy outcomes. This surveillance site has been described in detail elsewhere [19].

The study was a prospective open cohort study involving all children born at the Dabat HDSS during November 2009 and August 2011. Children were recruited at birth and followed until the age of one year.

Data were collected using a structured questionnaire adapted from the UNICEF Multiple Indicator Cluster Survey (MICS) questionnaire [20]. It was pre-tested in a population outside the HDSS area. Three field supervisors with previous experience supervised the day to day data collection process. Seventeen data collectors graduated from high school and with similar previous experience were used to collect the data. A Five day training was given to all field workers on the data collection tools and study procedures prior to the commencement of the study. Data collectors and supervisors were assisted during the field work by local informants who were residents of the study villages. The local informants were recruited to report pregnancy related events to the data collectors irrespective of the pregnancy outcome. Pregnant women were identified through the regular surveillance visits and by village informants. Regular supervision was conducted by the principal investigator throughout the study period.

Observation time for each infant, started from birth or from the time of migration into the area and ended at the time of death or at the time of migration out of the surveillance area.

Double data entry was done using Epi-Info for windows Version 3.5. After completing data validation and cleaning, the data were transferred to STATA Version 11 software for analysis.

Both vicariate and multivariate Poisson regression analyses were used to calculate incidence rate ratios along with their 95% CI and assess interactions between different variables. Probability of surviving was estimated by Kaplan-Meier (K-M) survival estimates. Incidence rates were expressed per 1,000 person years of observation.

Two -level random effects Poisson regression model was initially fitted to account for the intra cluster correlation of infant born from mothers in the same household and those born from households in the same village which showed evidence of clustering only at the household level. Thus, the final model was adjusted only for clustering at the household level.

We had assessed the effect of important confounding variables using five different models. In each of the models residence was taken as main exposure variable. Model 1 socioeconomic characteristic; model 2 nutritional; model 3 environmental; model 4 behavioral; and model 5 maternal biological factors were considered in the analysis. In the final model, residence as main exposure variable and all other covariates from each model with a p-value of less than 0.05 was included in the final analysis.

### Ethical considerations

The study was ethically approved by the Research and Ethics Committee of the University of Gondar with the Ref. No. RPO 55/338/2001. Participation was completely voluntary bases (Ethical form attached). Consent was sought from each individual participant before the commencement of each interview. Since majority of women are illiterate, the ethics committee approved to secure verbal consent from the study participants in their own language. No names were attached to the questionnaire. The study subjects were only identified by their individual unique identifier registration numbers and were also referenced in case of each follow up. Permission to conduct the study in the local area was obtained from the local administrations to conduct the study. Only individuals who had been consented to participate were included in the study. No one was coerced in any way to participate. Lastly the participants were also informed that they had the right to abstain from the study or to withdraw at any point. The participants were also assured about the confidentiality of the data.

## Results

The study followed a total of 1752 live births constituting 1472 person-year of follow-up. The overall infant mortality rate was 88.29 per 1000 person-years (95% CI: 74.3, 104.9). The average maternal age at birth of the index child was 27.76 years. Majority (82.99%) of the infants were born in rural areas and from mothers who were illiterate (72. 71%), and housewives (89.13%).

The multivariate Poisson regression analysis revealed exclusive breastfeeding as the strongest predictor of infant mortality; children who were not exclusively breastfed were about 8 times more likely to die [(IRR = 7.86, 95% CI: (5.11, 12.10)]. Other factors associated with infant mortality include rural residence [(IRR = 2.33, 95%CI: (1.3, 5.3)], maternal illiteracy [(IRR = 6.48, 95%CI: (2.71, 15.52)], children born other than married women [(IRR = 7.48, 95%CI: (4.26, 13.17)] infant living in thatched roof houses [(IRR = 2.12, 95%CI: (1.42, 3.16)], mothers who didn’t wash their hands after visiting a toilet and before feeding a child [(IRR = 4.61, 95%CI: (2.24, 9.48)], birth interval less than 24 months [(IRR = 2.79, 95%CI: (1.88, 4.15)], mothers who hadn’t used any contraceptive methods [(IRR = 1.65, 95% CI: (1.02, 2.66)], initiated breast milk within 1–24 hours [(IRR = 2.96, 95% CI: (1.76, 4.96)], initiated breast milk after 24 hour [(IRR = 4.84, 95% CI: (2.94, 7.99)], and children who were not given gruel as a complementary food at the age of six months [IRR = 14.59, 95% CI: (9.39, 22.67)] have got increased risk of infant mortality (Table 1).

**Table 1:** **Multivariate Poisson regression final model in predicting in** **fant mor** **tality in northwest, Ethiopia, Nov.2009 to August 2011**

**Descriptions**	**Frequency (%)**		**CIRR 95% confidence interval**	**AIRR 95% confidence interval**
	**Deaths**	**Survived**		
**Residential area**				
Urban	11 (0.79)	1,387 (99.21)	1	1
Rural	119 (1.79)	6,701 (98.26)	2.23 (1.186 4.19)	2.33 ( 1.12 4.88)*
**Maternal marital status**				
Living in marriage	109 (1.41)	7,600 (98.59)	1	1
Single (never married, widowed & separated)	21 (4.13)	488 (95.87)	4.33 (2.47 7.60)	7.48 ( 4.26 13.17)*
**Maternal education**				
Illiterate	112 (1.87)	5,865 (98.13)	1.98 (0.89 4.37)	6.48 ( 2.71 15.52)*
Read & write/ primary	12 (1.05)	1,129 (98.95)	1.21(0.48 3.08)	1.38 (0.51 3.70)*
Secondary and above	6 (0.55)	1,094 (99.45)	1	1
**Wealth index**				
Lowest quintile	56 (2.04)	2,684 (97.96)	1.70 (1.10 2.65)	1.00 (0.59 1.52)
Medium quintile	41 (1.50)	2,699 (98.50)	1.26 (0.79 2.02)	0.84 (0.51 1.37)
Highest quintile	33 (1.21)	2,705 (98.79)	1	1
**Roof**				
Corrugated Iron sheet	49 (1.35)	3,570 (98.65)	1	1
Thatch or grass	81 (1.76)	4,518 (98.24)	1.65 (1.14 2.40)	2.12 ( 1.42 3.16)*
**Window**				
Yes	78 (1.34)	5,734 (98.66)	1	1
No	52 (2.16)	2,354 (97.84)	1.59 (1.10 2.28)	1.13 (0.78 1.63)
**Hand washing with soap at critical points**				
Yes	11 (0.73)	1,486 (99.27)	1	1
No	119 (1.77)	6,602 (98.23)	1.97 ( 0.98 3.99)	4.61 ( 2.24 9.48)*
**Birth interval**				
< 24 months	55 (2.38)	2,257 (97.62)	1.98 (1.38 2.85)	2.79 (1.88 4.15)*
>24 months	75 (1.27)	5,831 (98.73)	1	1
**Ever use of contraceptive methods**				
Yes	1	25 (1.20)	2,058 (98.80)	1
No	105 (1.71)	6,030 (98.29)	1.15 (0.70 1.87)	1.65 (1.02 2.66)*
A child given semi-solid as complementary food at six months				
Yes	32 (0.52)	6,126 (99.48)	1	1
No	98 (4.76)	1,962 (95.24)	5.16 (2.98 8.95)	14.59 ( 9.39 22.67)*
**Exclusive Breast feeding**				
Yes	29 (0.48)	5,976 (99.52)	1	1
No	101 (4.56)	2,112 (95.44)	10.37 (6.77 15.90)	7.86 ( 5.11 12.10)*
**Time of initiation of breast milk**				
Immediately 1- 24 hours	23 (0.58)	3,954 (99.42)	1	1
	44 (1.78)	2,431 (98.22)	2.98 (1.78 5.00)	2.96 (1.76 4.96)*
More than 24 hours	63 (3.57)	1,703 (96.43)	6.10 (3.69 10.06)	4.84 (2.94 7.99)*

*= Showing significant variables.

## Discussion and Conclusions

The study revealed a very high infant mortality rate in the study area. It also revealed that the strongest predictors of infant mortality are found to be breastfeeding and unhygienic practices of mothers in predominantly rural poor settings. The infant mortality rate observed in this study was very high when compared to that of the previous national and regional reports made by WHO, 2011[4] and EDHS, 2011[5]. The possible reason could be the difference in the study design and populations under the study cohort. Survey was likely to miss infant deaths due to either recall or cultural taboos related to reporting infant death, especially deaths near to birth [21,22]. The longitudinal nature of this study allowed capturing deaths as they occur and minimized the chance of under estimation.

Of the nutritional factors, early initiation of breast-feeding associated with reduced risk of infant mortality was consistent with the previous studies reported showing a strong relationship between infant mortality and early initiation of breast milk. The risk of infant death increases when breast milk is not initiated immediately after birth [13,14]. About 20% of all neonatal deaths could be prevented if all newborns were initiated breastfeeding within one hour of birth [16,21,22]. Early breastfeeding initiation reduces the risk of infectious diseases, which is one of the most common causes of death during the neonatal period [17]. Children who did not receive exclusive breastfeeding had also a high risk of dying in this study population consistent with previous studies [21].

Other factors that are known to negatively affect infant survival such as maternal illiteracy as shown in many other studies, is inversely associated with infant mortality. Women who were illiterate had the highest infant mortality rate as compared to those having secondary education and above. Similarly, various studies have supported the association and direct causal relationship of maternal education and infant mortality [7, 8, 22, 23]. The possible reason could be mothers with higher education might probably have better access to the utilization of health facilities [6,7,12] and higher income helps mothers to have the ability to purchase goods and services that in turn helps to improve infants’ health [7,8,23].

Regarding the geographical position of households, the study shows that rural areas have a higher rate of the IMR than the urban areas. Many scholars have observed a significant difference between urban and rural infant mortality [6,10]. This could be due to the fact that urban areas had good infrastructures and better access to health facilities than the rural ones [2,6,10].

Previous studies indicated that marital status is one of the important determinants for infant mortality; married women reduced risk of infant mortality when compared in relation to that of divorced/widowed and single category [9,10,24]. In line with this, the finding of this study indicates that infant born to married women plays an important significant role 7.86 (95% CI: 4.26, 13.17) for the reduction of infant mortality in comparison to infants born from other categories (single, widowed and separated). This might be due to socioeconomic factors, traditions and the lifestyle of the single, widowed and separated mothers [9,10,24].

Among maternal biological factors, birth interval with previous child has a strong relationship with infant mortality for the index child. The result indicates that the infant mortality rate is found to be the highest for infants having less than 24 months of birth interval with the previous child and lowest for the infant whose birth interval was above 24 months. This finding has conformed with the other studies that the length of the birth interval is positively correlated with the survival of the infant mortality. Short birth interval increases the risk of infant mortality due to physiological and nutritional depletion of the mothers which relate the mothers exposed to pregnancy complication [25,26].

The research also indicates that children born from mothers who have not any experience of family planning services have 65% increased risk of infant mortality. The possible explanation is that women who have the experience of family planning services might reduce their reproductive behavior risks, prolong birth intervals, lower fertility [11], result in reduced infant mortality. The possible reason could be family planning (FP) methods are important for reproductive health and well-being and is one of the most cost-effective public health interventions available in developing countries to lower the rates of infant mortality (IMR) and to promote economic growth [27-29]. Family planning also helps to reduce the number of high-risk pregnancies, thereby, reducing the high levels of maternal and infant mortality associated with risky pregnancies [11,28].

Infants born from mothers who did not have the experience of using soap for washing hands before feeding a child and after visiting a toilet had 2.11 (95% CI: 1.04 to 4.28) times higher risk of dying than those who practiced using soap. This might be directly related to hygienic practices that reduce contamination and infections. Mortality due to infection is likely to make up a greater proportion of deaths among infants with these characteristics. The trend seen here is consistent with the hypothesis that hand-washing reduces the overall exposure of the newborns to potentially invasive pathogens and the impacts on mortality due to infection [10,30].

Although the findings offer important insights about risk factors about infant mortality, there are limitations that one should bear in mind when interpreting the results such as the absences of complete birth weight data since most of the deliveries were made at home.

These findings have important implications for infant mortality, especially, for neonatal health programs and policy making. Countries with the largest reductions in infant mortality have had substantial improvements in most of the factors that might be used to explain these changes. In some developing countries like Ethiopia, infant mortality has not decreased to the desired level in order to meet the millennium development goal. In part, these issues can be explained by the factors such as promotion of early initiation and exclusive breast feeding, maternal education, hand washing with soap at critical points, birth interval and use of contraceptive methods. Three hundred eighty four percent of infant deaths could be decreased if all infants were breastfed before 24 hours and 196% if breastfeeding was started within the first hour to 24 hours after birth. In addition, when children are not exclusively breast fed, the risk of infant death will increase by 686%. Given these important consequences of breast-feeding on birth intervals and reducing infant mortality, the subject of the determinants of breast-feeding practices should get increasing interest. It is suggested that breastfeeding-promotion programs in predominantly rural settings should place considerable emphasis on early initiation of breastfeeding as well as promoting exclusive breastfeeding. It is also noted that (We note that) the policy implications here are likely to have relevance for other developing countries provided that relevant cultural and socioeconomic conditions are similar to those of northwest Ethiopia.

Infant mortality in the northwest part of the country is still very high. Exclusive breast feeding is the strongest predictor of infant survival in this predominantly rural setting where hygienic standards are poor. Supporting mothers to exclusively breast feeding their children, which is cost effective, safe and feasible strategy, can help reduce infant mortality in the study setting towards the made in achieving the MDGs by 2015.k.
